# Cortical transcriptomic dysregulation in Autism spectrum disorder: A conceptual synthesis

**DOI:** 10.1016/j.ibneur.2026.06.004

**Published:** 2026-06-06

**Authors:** Ruslan Kurmashev

**Affiliations:** Department of Biological Sciences, Munster Technological University, Bishopstown, Cork T12 P928, Ireland

**Keywords:** Autism spectrum disorder, Postmortem cortex, Cortical transcriptomics, Synaptic programs, Immune-glial programs, RNA regulatory processes

## Abstract

Autism spectrum disorder (ASD) is a heterogeneous neurodevelopmental condition in which human post-mortem cortical transcriptomic studies have identified recurrent but context-sensitive molecular abnormalities. This narrative review synthesises RNA sequencing (RNA-seq) evidence from ASD cortex and organises recurrent findings into three interacting cortical domains: synaptic/neuronal programmes, immune-glial programmes, and RNA-regulatory processes. Across bulk cortical datasets, the most reproducible signals include relative downregulation of neuronal and synaptic expression programmes together with upregulation of glial-associated and neuroimmune signatures, whereas transcript-level analyses further implicate alternative splicing, microexon dysregulation, differential transcript usage, and isoform-level perturbation. Single-nucleus and related cell-resolved approaches localise these abnormalities to defined neuronal and glial populations, while long-read strategies strengthen inference on transcript structure and isoform complexity. Within this framework, mitochondrial-related findings are best interpreted as a linked bioenergetic component rather than an equally established standalone transcriptomic domain. The review also summarise the principal interpretive constraints of cortical ASD transcriptomics, including region specificity, developmental stage, cohort heterogeneity, cell-type composition, post-mortem tissue quality, and cross-platform comparability.

## Highlights

• Human postmortem cortex reveals recurrent ASD transcriptomic dysregulation.
• Cortical ASD signals cluster into synaptic/neuronal and immune-glial programs.
• RNA-regulatory disruption includes splicing, microexons, and isoform changes.
• Mitochondrial signals are best treated as linked bioenergetic components.
• Bulk, single-nucleus, and long-read RNA-seq provide complementary inference.

## 1 Introduction

Autism spectrum disorder (ASD) is a heterogeneous neurodevelopmental condition characterised by persistent differences in social communication and interaction alongside restricted, repetitive patterns of behaviour, interests, or sensory features (American Psychiatric Association, 2013, Lord et al., 2020). Symptoms typically emerge in early childhood and persist across the lifespan (Lord et al., 2020). According to the latest CDC Autism and Developmental Disabilities Monitoring Network report, ASD was identified in 32.2 per 1000 8-year-old children in the United States in 2022, corresponding to approximately 1 in 31 children, and remained more frequently identified in boys than girls (Shaw et al., 2025).

Human post-mortem cerebral cortex provides a particularly informative substrate for interpreting ASD biology because it offers direct access to molecular and cellular abnormalities within the tissue most consistently interrogated by transcriptomic and broader neuropathological studies of the disorder (Fetit et al., 2021). Across cortical transcriptomic datasets, recurrent abnormalities have included relative downregulation of neuronal and synaptic gene-expression programmes together with upregulation of glial and immune-related signatures, although their magnitude and distribution vary by region, developmental stage, cohort composition, and analytical framework (Voineagu et al., 2011, Gupta et al., 2014, Parikshak et al., 2016, Gandal et al., 2022). This cortex-centred emphasis is also consistent with broader post-mortem evidence for spatially heterogeneous and layer-specific cortical abnormalities in ASD, including focal cortical disorganisation and altered neuronal or glial cell distribution in specific cortical layers (Stoner et al., 2014, Falcone et al., 2021, Fetit et al., 2021). Cortical transcriptomics is therefore most informative not as a search for a single invariant molecular lesion, but as a means of identifying recurring biological programmes within a heterogeneous condition.

Recent work has also shown that gene-level differential expression alone does not exhaust the transcriptomic architecture of ASD cortex. In addition to broad shifts in cortical gene-expression modules, studies have implicated altered regional patterning, alternative splicing, differential transcript usage, and isoform-level dysregulation, indicating that ASD-related abnormalities extend to the level of RNA processing and transcript structure (Parikshak et al., 2016, Gandal et al., 2018). Cell-resolved approaches have refined this picture further by localising dysregulation to specific neuronal and glial populations, while newer single-cell analyses have identified molecular cascades and cell type-specific signatures that link ASD-associated risk to defined cortical cellular states (Velmeshev et al., 2019, Wamsley et al., 2024). Neuron-focused transcriptomic work has similarly suggested that inflammatory, synaptic, mitochondrial, and splicing-related abnormalities can co-occur within discrete cortical cellular compartments rather than appearing only as bulk-tissue averages (Zhang et al., 2023).

Against this background, the present review offers a conceptual synthesis of cortical transcriptomic dysregulation in ASD. Rather than treating any single pathway as definitive, it organises recurrent cortical findings around interacting synaptic/neuronal, immune-glial, and RNA-regulatory programmes, with mitochondrial and broader bioenergetic signals considered linked but less consistently established components of this framework (Gupta et al., 2014, Parikshak et al., 2016, Gandal et al., 2018, Gandal et al., 2022, Schwede et al., 2018). The review therefore prioritises human post-mortem cortical evidence, introduces transcript-level regulation early as a central interpretive theme, and treats peripheral transcriptomic findings only as limited contextual comparisons.

## 2 Conceptual framing of cortical transcriptomic dysregulation in ASD

In the present review, recurrent findings from human post-mortem ASD cortex are organised into three interacting transcriptomic domains rather than interpreted as isolated signals. The first comprises synaptic and broader neuronal programmes related to neuronal communication, synaptic organisation, and cortical circuit function. The second comprises immune-glial programmes, including glial and neuroimmune signatures detected within cortical tissue. The third comprises RNA-regulatory processes, encompassing alternative splicing, microexon regulation, differential transcript usage, isoform-level dysregulation, and related disturbances in transcript processing. Considered together, these domains provide a more precise framework for interpreting cortical ASD transcriptomics than any single pathway viewed in isolation (Voineagu et al., 2011, Gupta et al., 2014, Parikshak et al., 2016, Gandal et al., 2022).

Positioning RNA-regulatory processes centrally is important because ASD-related cortical dysregulation is not confined to shifts in total gene abundance. Transcript-level studies have implicated misregulated neuronal microexons, alternative splicing, altered regional transcript patterning, differential transcript usage, and isoform-level perturbation, indicating that RNA processing and transcript structure form a substantive layer of cortical molecular dysregulation rather than a secondary technical detail (Irimia et al., 2014, Parikshak et al., 2016, Gandal et al., 2018). More resolved neuronal analyses have strengthened this interpretation by showing that alternative splicing and related transcriptomic abnormalities can be detected within specific cortical cellular contexts and may shape how broader synaptic and immune-glial signals are expressed (Zhang et al., 2023, Wamsley et al., 2024).

These domains should also not be understood as independent layers of pathology. Cortical studies indicate that immune-glial activation may occur alongside altered neuronal activity-dependent or synaptic programmes, whereas cell-resolved analyses localise ASD-associated dysregulation to specific neuronal and glial populations rather than a uniform cortical state (Gupta et al., 2014, Velmeshev et al., 2019, Wamsley et al., 2024). Within this framework, mitochondrial and broader bioenergetic findings are best interpreted as linked components of cortical dysregulation: synaptic dysfunction may alter energetic demand, and neuron-specific transcriptomic work suggests that inflammatory, synaptic, mitochondrial, and splicing-related abnormalities can co-occur within cortical cellular compartments (Zhang et al., 2023). This reading is also consistent with evidence that mitochondrial-related transcriptional changes correlate with downregulated synaptic programmes in post-mortem autism cortex, while still supporting more cautious weighting of mitochondrial findings than of the three core transcriptomic domains defined here (Schwede et al., 2018).

**Fig. 1:**
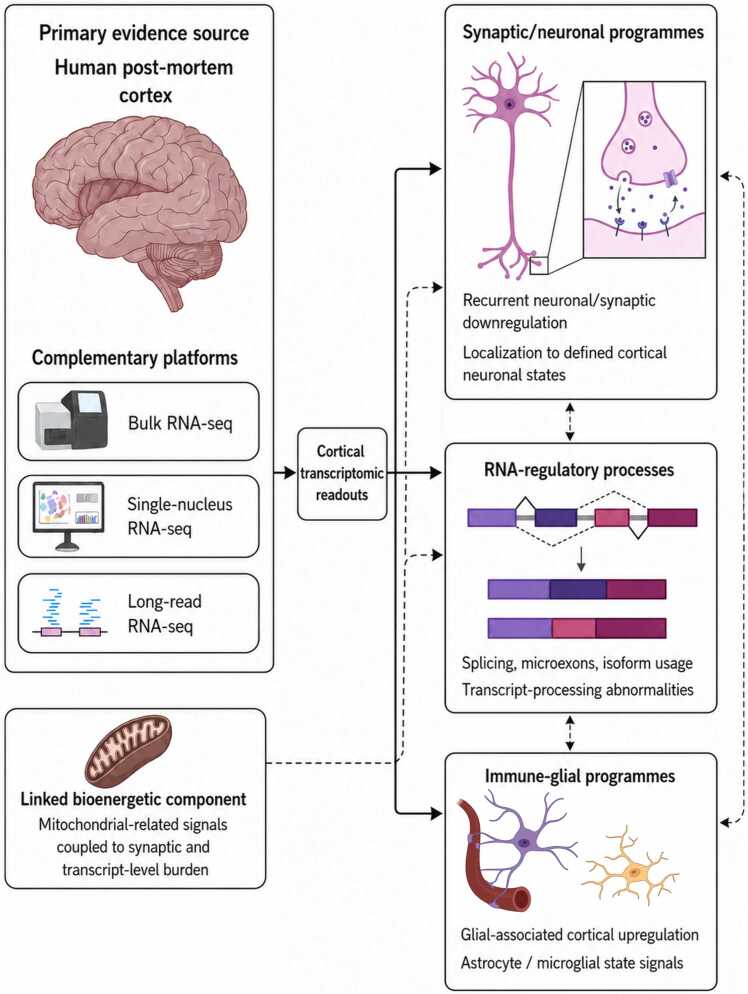
Cortical transcriptomic domains in Autism spectrum disorder. Human post-mortem cortex provides the primary evidence base for this framework, which organises recurrent findings into synaptic/neuronal, immune-glial, and RNA-regulatory domains, with mitochondrial-related signals represented as a linked bioenergetic component.

The following sections examine each domain in turn, beginning with synaptic and neuronal dysregulation. Fig. 1 summarises the conceptual framework used in this review, organising recurrent cortical ASD transcriptomic findings into synaptic/neuronal, immune-glial, and RNA-regulatory domains, while positioning mitochondrial-related signals as a linked bioenergetic component.

### 2.1 Synaptic and neuronal dysregulation

Among recurrent cortical transcriptomic findings in ASD, altered synaptic and broader neuronal programmes are among the most consistently observed. Early post-mortem cortical transcriptomic work identified a disrupted neuronal co-expression module linked to synaptic and neuronal function, together with attenuation of the regional expression differences that ordinarily distinguish frontal and temporal cortex (Voineagu et al., 2011). Subsequent cortical studies strengthened this pattern by reporting dysregulation of neuronal activity-dependent genes, broad changes in synaptic and neuronal expression programmes, altered regional transcriptomic organisation, and downregulation of synaptic gene sets across wider cortical sampling frameworks (Gupta et al., 2014, Parikshak et al., 2016, Gandal et al., 2022). Independent integrative work further showed that autism-associated cortical thickness differences are enriched for synaptic and transcriptionally downregulated genes that overlap strongly with post-mortem cortical dysregulation, supporting the anatomical relevance of this transcriptomic signal beyond any single cohort (Romero-Garcia et al., 2019). The strength of this signal lies less in any single candidate gene than in the recurrence of programme-level neuronal and synaptic dysregulation across independent cortical transcriptomic analyses.

More recent single-cell and single-nucleus studies indicate that synaptic and neuronal dysregulation is not distributed uniformly across cortex but is enriched within defined neuronal populations. In particular, upper-layer excitatory neuronal populations have been repeatedly implicated, and newer single-cell analyses further connect ASD-associated molecular cascades to specific cortical cellular states rather than to a diffuse tissue-wide abnormality (Velmeshev et al., 2019, Wamsley et al., 2024). This interpretation is broadly compatible with convergent post-mortem cortical evidence for laminar and inhibitory-microcircuit abnormalities, including layer-specific glia-neuron imbalance within transcriptomically disorganised cortical patches and reduced chandelier-cell-related inhibitory elements in prefrontal cortex (Rabelo et al., 2023, Ariza et al., 2018, Amina et al., 2021). Neuron-focused transcriptomic analyses also suggest that altered neuronal activity-related signatures can co-occur with neural-immune, stress-related, and transcript-processing abnormalities within neuronal compartments, underscoring that synaptic and neuronal dysregulation should be interpreted within a broader but still cortex-centred molecular context (Zhang et al., 2023). Interpretation nevertheless remains conditioned by region, developmental stage, cohort heterogeneity, and the sensitivity of bulk measurements to cell-type composition. These neuronal and synaptic signals are complemented by recurrent cortical immune-glial changes, which form the second major domain of the present framework.

### 2.2 Immune-glial programmes in ASD cortex

Alongside synaptic and neuronal dysregulation, ASD cortical transcriptomic studies recurrently report upregulation of immune-related and glial-associated programmes. Early cortical network-level analyses identified non-neuronal co-expression modules linked to glial and immune-related function, while subsequent transcriptomic studies strengthened this observation by reporting dysregulation of innate immune response genes and broader immune-glial signatures within post-mortem cortex (Voineagu et al., 2011, Gupta et al., 2014). Later large-scale cortical analyses further supported the recurrence of this signal across regions and cohorts, indicating that immune-glial changes constitute a reproducible transcriptomic domain of ASD cortex rather than a purely peripheral observation (Parikshak et al., 2016, Gandal et al., 2022). This transcriptomic pattern is broadly compatible with direct post-mortem cortical evidence for altered glial marker expression and astrocytic abnormalities in autism, including reduced astrocyte number with increased activation state in prefrontal cortex (Edmonson et al., 2014, Vakilzadeh et al., 2022). The term immune-glial is used here as a transcriptomic descriptor of glial-associated and neuroimmune programmes, not as a claim of uniform inflammatory pathology across all cortical regions or ASD cases.

Cell-resolved studies refine this picture by localising ASD-associated dysregulation to defined cortical cellular states rather than to a nonspecific tissue-wide inflammatory background. Single-nucleus analyses implicated microglial molecular states alongside upper-layer excitatory neuronal populations, and newer single-cell studies similarly connected ASD-associated molecular cascades to specific cortical cell types (Velmeshev et al., 2019, Wamsley et al., 2024). This state-based interpretation is also consistent with human cortical neuropathology showing increased microglial density and activation in dorsolateral prefrontal cortex, abnormal microglial-neuronal spatial organisation, and developmental microglial priming in temporal cortex (Morgan et al., 2010, Morgan et al., 2012, Lee et al., 2017). Neuron-focused analyses further suggest that neural-immune or stress-related transcriptomic features may also be detectable within neuronal compartments, underscoring that immune-glial programmes intersect with other cortical domains rather than operating in isolation (Zhang et al., 2023). By contrast, peripheral blood transcriptomic studies may offer only limited contextual comparison because they are strongly shaped by leukocyte composition, developmental stage, and clinical-state confounding; accordingly, they should not be treated as direct proxies for cortical glial mechanisms (Li et al., 2023, Tomaiuolo et al., 2023). These cortical immune-glial programmes intersect closely with transcript-level dysregulation, including splicing, isoform usage, and broader RNA-regulatory processes, which form the third domain of the present framework.

### 2.3 RNA-regulatory processes in ASD cortex

ASD cortical dysregulation is not confined to shifts in total gene abundance. Across post-mortem cortical studies, recurrent transcript-level abnormalities include alternative splicing changes, attenuation of normal regional transcript patterning, microexon dysregulation, isoform-level perturbation, differential transcript usage, and altered long noncoding RNA expression, indicating that RNA processing contributes substantively to the molecular architecture of ASD cortex (Voineagu et al., 2011, Irimia et al., 2014, Parikshak et al., 2016, Gandal et al., 2018, Ziats and Rennert, 2013). Large cross-regional analyses further show that broad cortical gene-expression dysregulation coexists with transcript-level disruption, reinforcing that RNA-regulatory processes should be interpreted as a central domain of ASD cortical transcriptomics rather than as a secondary technical feature of RNA-seq (Gandal et al., 2022).

Newer studies refine this domain by showing that transcript-processing and isoform-related abnormalities can be resolved within defined cortical cellular contexts. Isoform-network analyses and differential transcript usage have already shown that transcript-level dysregulation is structured rather than diffuse (Gandal et al., 2018), and more recent single-cell work has linked ASD-associated molecular cascades to specific cortical cell types (Wamsley et al., 2024). This broader interpretation is also consistent with mechanistic work showing that neuronal splicing regulators such as RBFOX1 coordinate both splicing and transcriptional networks during human neuronal development, although such studies provide contextual support rather than direct ASD cortical evidence (Fogel et al., 2012). Neuron-focused analyses further indicate that transcript-processing abnormalities may coexist with altered neuronal activity and stress- or neural-immune-related states within neuronal compartments, supporting the view that RNA-regulatory disruption may shape how both synaptic and immune-glial programmes are expressed in cortex (Zhang et al., 2023).

Within this framework, mitochondrial and broader bioenergetic findings are best interpreted as linked components of cortical dysregulation rather than as an equally established standalone domain. The strongest support for this interpretation comes from evidence that mitochondrial-related downregulation correlates closely with downregulation of synaptic transmission-related programmes in post-mortem autism cortex, consistent with a coupled bioenergetic-synaptic burden rather than a separate mitochondrial transcriptomic axis of equal evidential weight (Schwede et al., 2018). More limited region-specific studies have reported nominal mitochondrial-related expression differences, but such findings did not remain robust after multiple-testing correction and therefore require independent replication (Anitha et al., 2012). Overall, mitochondrial findings remain relevant to the present framework, but their support is more limited and heterogeneous than that for RNA-regulatory processes themselves. These transcript-level considerations also help explain why different RNA-seq platforms capture complementary but non-identical layers of ASD cortical dysregulation.

### 2.4 Transcriptomic approaches and interpretive constraints

Different RNA-seq approaches should not be viewed as interchangeable in ASD cortical research, because they capture distinct but complementary layers of transcriptomic dysregulation. This distinction is especially important in the present framework, in which synaptic/neuronal programmes, immune-glial states, and RNA-regulatory processes are inferred at different levels of biological resolution. Accordingly, the principal value of each platform lies not only in sequencing depth or throughput, but also in the type of inference it can support (Conesa et al., 2016, Gandal et al., 2022, Pardo-Palacios et al., 2024).

Bulk RNA-seq remains the strongest approach for identifying tissue-level programmes across post-mortem cortex, including coordinated neuronal/synaptic downregulation, immune-glial upregulation, and broad transcriptomic reorganisation across cortical regions (Parikshak et al., 2016, Gandal et al., 2022). Its major limitation is that estimates are averaged across mixed cell populations and are therefore sensitive to shifts in cellular composition, tissue quality, and region-specific sampling.

Single-cell and single-nucleus RNA-seq provide a different level of inference by localising dysregulation to defined cortical cell populations and states rather than to bulk tissue as a whole. In ASD cortex, these approaches have helped resolve signals in upper-layer excitatory neurons, microglial states, and other cellular compartments, thereby refining how synaptic, immune-glial, and stress-related programmes are interpreted (Velmeshev et al., 2019, Wamsley et al., 2024). Their main constraints are sample availability, technical complexity, tissue handling requirements, and cross-cohort comparability.

Long-read RNA-seq is particularly relevant to the RNA-regulatory domain because it improves structural resolution of full-length transcripts, isoforms, and previously unannotated transcript variants. Its main contribution is therefore not broad programme discovery, but stronger transcript-structure and isoform inference, which can complement short-read findings on splicing and transcript usage. At the same time, quantification remains constrained by throughput, platform-specific error profiles, and pipeline dependence (Pardo-Palacios et al., 2024).

Taken together, these approaches are complementary but not interchangeable: bulk RNA-seq is best suited to programme-level discovery across cortex, sc/snRNA-seq to cell-type and cell-state localisation, and long-read RNA-seq to transcript-structure and isoform resolution. Interpretation across platforms nevertheless remains conditioned by region specificity, developmental stage, cohort heterogeneity, tissue quality, and analytic workflow (Conesa et al., 2016, Gandal et al., 2022, Velmeshev et al., 2019, Wamsley et al., 2024, Pardo-Palacios et al., 2024). Table 1 summarizes the biological resolution, main interpretive contribution, principal limitations, and representative sources for transcriptomic approaches relevant to cortical autism spectrum disorder studies.

**Table 1:** Transcriptomic approaches relevant to cortical ASD studies: inferential strengths and main limitations.

**Approach**	**Biological resolution**	**Primary contribution to the present framework**	**Main limitation in ASD cortex**	**Representative source(s)**
**Bulk RNA-seq**	Mixed-cell tissue level	Identifies programme-level cortical dysregulation, including recurrent synaptic/neuronal and immune-glial shifts across regions.	Sensitive to cell-type composition, tissue quality, and region-specific sampling.	Parikshak et al., 2016; Gandal et al., 2022
**Single-nucleus RNA-seq**	Cell-type and cell-state level	Localises dysregulation to defined neuronal and glial populations, refining interpretation of synaptic and immune-glial programmes.	Smaller cohorts, technical complexity, tissue handling constraints, and limited cross-study standardisation.	Velmeshev et al., 2019; Wamsley et al., 2024
**Long-read RNA-seq**	Transcript and isoform structure	Refines RNA-regulatory hypotheses through full-length transcript and isoform resolution.	Lower throughput, quantification uncertainty, and pipeline dependence.	Pardo-Palacios et al., 2024

Note. Biological resolution refers to the principal inferential level supported by each approach in human post-mortem cortical ASD studies.

### 2.5 Illustrative transcriptomic signals in ASD cortex

The examples summarized in Table 2 are intended as illustrative cortical transcriptomic signals rather than diagnostic markers. They are selected to show how the present framework is instantiated in human ASD cortex across synaptic/neuronal, immune-glial, and RNA-regulatory domains, with a separate row for linked bioenergetic coupling where direct cortical transcriptional support exists.

**Table 2:** Illustrative cortical transcriptomic signals supporting the conceptual framework in ASD.

**Domain**	**Illustrative cortical signal**	**Transcriptomic level**	**Interpretive relevance**	**Representative source(s)**
**Synaptic/ neuronal**	Programme-level downregulation of neuronal and synaptic expression modules	Bulk cortical programme level	Anchors the synaptic/neuronal domain as one of the most recurrent transcriptomic patterns in ASD cortex.	Voineagu et al., 2011; Parikshak et al., 2016; Gandal et al., 2022
**Immune-glial**	Upregulation of glial-associated and immune-related cortical programmes	Bulk cortical programme level	Supports the immune-glial domain as a cortical transcriptomic pattern rather than a peripheral inflammatory proxy.	Gupta et al., 2014; Gandal et al., 2022
**Synaptic/neuronal and RNA-regulatory**	Alternative splicing and regional transcript-pattern disruption	Alternative splicing / exon-level regulation	Shows that transcript-level dysregulation is embedded within broader cortical transcript organisation rather than occurring as an isolated technical feature.	Voineagu et al., 2011; Parikshak et al., 2016
**RNA-regulatory**	Microexon dysregulation	Microexon / fine-grained splicing regulation	Provides strong evidence that ASD cortical dysregulation extends to highly specific RNA-processing programmes.	Irimia et al., 2014; Quesnel-Vallières et al., 2016
**RNA-regulatory**	Isoform-level dysregulation and differential transcript usage	Isoform / transcript-level regulation	Supports RNA-regulatory processes as a central domain beyond total gene-expression shifts.	Gandal et al., 2018; Parikshak et al., 2016
**RNA-regulatory**	Altered long noncoding RNA expression	Noncoding transcript regulation	Extends the RNA-regulatory domain beyond coding-gene abundance and splicing to noncoding transcript dysregulation in ASD cortex.	Ziats and Rennert, 2013; Parikshak et al., 2016
**Synaptic/neuronal and immune-glial**	Cell-type-resolved neuronal and glial dysregulation	Cell-type and cell-state level	Refines bulk cortical findings by localising dysregulation to defined neuronal and glial populations.	Velmeshev et al., 2019; Wamsley et al., 2024
**Linked bioenergetic component**	Coupled mitochondrial-related and synaptic downregulation	Linked programme-level bioenergetic signal	Retains mitochondrial findings within the framework while weighting them as a linked component rather than an equally established standalone domain.	Schwede et al., 2018; Anitha et al., 2012
**RNA-regulatory**	Neuron-specific transcript-processing and stress-related abnormalities	Neuron-focused transcript-processing context	Strengthens the link between RNA-regulatory abnormalities and defined neuronal states in ASD cortex.	Zhang et al., 2023

Note. Signals are illustrative rather than diagnostic and are restricted to human cortical transcriptomic evidence, except where linked bioenergetic coupling is stated explicitly.

### 2.6 Key limitations and priorities

Interpretation of cortical ASD transcriptomics remains limited by region specificity, developmental stage, clinical heterogeneity, and the dependence of bulk cortical datasets on cell-type composition and post-mortem tissue quality. Although single-nucleus and long-read approaches substantially improve cellular and transcript-structural resolution, they remain constrained by sample availability, technical complexity, throughput, and cross-study comparability. Future priorities therefore include replication across independent cortical cohorts and regions, closer integration of bulk, single-nucleus, and isoform-aware approaches, and continued caution when relating peripheral transcriptomic signals to cortical immune-glial states (Parikshak et al., 2016, Velmeshev et al., 2019, Gandal et al., 2022, Pardo-Palacios et al., 2024).

## 3 Conclusion

Human post-mortem cortical transcriptomic studies in ASD most consistently support recurrent downregulation of synaptic/neuronal programmes, upregulation of immune-glial signals, and disruption of RNA-regulatory processes, rather than a single invariant molecular signature. These findings are best interpreted as interacting cortical domains, with mitochondrial-related signals retained as a linked bioenergetic component rather than an equally established standalone domain. Future progress will depend on replication across cortical regions and developmental stages and on closer integration of bulk, single-nucleus, and isoform-aware approaches.

## CRediT authorship contribution statement

**Ruslan Kurmashev:** Writing – original draft, Validation, Resources, Methodology, Investigation, Data curation, Conceptualization.

## Ethics

This manuscript is a narrative review and does not report any new studies involving human participants or animals performed by the author. Therefore, ethics approval and informed consent are not applicable.

## Informed consent

Not applicable.

## Ethical approval

This article is a narrative review and does not involve human participants, personal data, or new experimental procedures. Ethical approval was therefore not required.

## Declaration of Generative AI and AI-assisted technologies in the manuscript preparation process

During the preparation of this manuscript, the author used ChatGPT (version 5.5, «thinking» model) solely for language-related assistance, including grammatically accurate translation, lexical refinement, and adaptation of the text to academic English. All translated and edited text was carefully reviewed and verified by the author and was confirmed to be fully consistent with the original content and the research conducted by the author. The study design, data analysis, interpretation of results, and all scientific conclusions were performed independently by the author, who takes full responsibility for the content of the published article.

## Funding

This research received no specific grant from any funding agency, commercial or not-for-profit sectors.

## Declaration of Competing Interest

The author declares that there are no conflicts of interest.
